# Synchronous firing of dorsal horn neurons at the origin of dorsal root reflexes in naïve and paw-inflamed mice

**DOI:** 10.3389/fncel.2022.1004956

**Published:** 2022-09-23

**Authors:** Javier Lucas-Romero, Ivan Rivera-Arconada, Jose A. Lopez-Garcia

**Affiliations:** Departamento de Biología de Sistemas, Universidad de Alcalá, Alcalá de Henares, Spain

**Keywords:** spinal cord, dorsal horn, dorsal root reflexes, spontaneous activity, primary afferent depolarization, inflammation, pain, central sensitization

## Abstract

Spinal interneurons located in the dorsal horn induce primary afferent depolarization (PAD) controlling the excitability of the afferent’s terminals. Following inflammation, PAD may reach firing threshold contributing to maintain inflammation and pain. Our aim was to study the collective behavior of dorsal horn neurons, its relation to backfiring of primary afferents and the effects of a peripheral inflammation in this system. Experiments were performed on slices of spinal cord obtained from naïve adult mice or mice that had suffered an inflammatory pretreatment. Simultaneous recordings from groups of dorsal horn neurons and primary afferents were obtained and machine-learning methodology was used to analyze effective connectivity between them. Dorsal horn recordings showed grouping of spontaneous action potentials from different neurons in “population bursts.” These occurred at irregular intervals and were formed by action potentials from all classes of neurons recorded. Compared to naïve, population bursts from treated animals concentrated more action potentials, had a faster onset and a slower decay. Population bursts were disrupted by perfusion of picrotoxin and held a strong temporal correlation with backfiring of afferents. Effective connectivity analysis allowed pinpointing specific neurons holding pre- or post-synaptic relation to the afferents. Many of these neurons had an irregular fast bursting pattern of spontaneous firing. We conclude that population bursts contain action potentials from neurons presynaptic to the afferents which are likely to control their excitability. Peripheral inflammation may enhance synchrony in these neurons, increasing the chance of triggering action potentials in primary afferents and contributing toward central sensitization.

## Introduction

Primary afferent depolarization (PAD) is the mechanism underlying presynaptic inhibition as first described in the ventral horn of the spinal cord (Frank and Fuortes, 1957; Eccles et al., 1961). Classical studies also determined that PAD is caused by release of GABA from presynaptic neurons. Primary afferents have abnormally high concentrations of Cl^–^ ions, presumably due to the activity of the chloride transporter NKCC1 (Rocha-González et al., 2008). Activation of GABA_A_ receptors causes an outflow of Cl^–^, which leads to PAD (Price et al., 2009).

Primary afferent depolarization occurs in the dorsal horn as well, where it may serve as a mechanism of sensory modulation (Boyle et al., 2019). PAD in nociceptive afferents is a plastic phenomenon that can be enhanced by peripheral inflammation but there is no evidence that other forms of injury produce such effects (Guo and Hu, 2014). Under inflammatory conditions PAD appears to be enhanced reaching action potential threshold to produce dorsal root reflexes (DRR) or backfiring in primary afferents (Lin et al., 2000). Antidromic action potentials traveling through nociceptive afferents lead to peripheral release of peptides and the generation of neurogenic inflammation (Willis, 1999). In addition, action potentials generated close to the afferent terminals may propagate orthodromically contributing to enhance excitability in nociceptive systems (Cervero et al., 2003). It has been proposed that under pathological conditions, an increased function of the chloride transporter NKCC1 may increase even further the intracellular concentration of Cl^–^ ions, enhancing the drive for depolarization and facilitating the firing of action potentials, perhaps with the mediation of calcium channels (Price et al., 2009). However, computer modeling predicts that other variables are required to turn PAD into trains of action potentials (Takkala et al., 2016).

Little is known about the circuitry involved in the generation of PAD although summation of presynaptic inputs was envisaged in early studies (Eccles et al., 1963). Chen et al. (1993) reported synchronous activity in dorsal roots from different segments, which persists in the isolated spinal cord. This activity has been related to the firing of superficial dorsal horn neurons connected to the Lissauer tract (Lidierth and Wall, 1998). Other groups located the generators of this activity in deeper or more superficial lamina (Jankowska et al., 1981; García et al., 2004). All these investigations on PAD and DRR mechanisms have used functional approaches based on single cell and field potential electrophysiological techniques. Therefore, the circuitry underlying these oscillations has been hypothesized (García et al., 2004; Chávez et al., 2012) but not directly observed.

Previous studies in our laboratory used electrode arrays to analyze spontaneous activity in small populations of superficial dorsal horn neurons (Roza et al., 2016; Lucas-Romero et al., 2018). We observed that some of them fired in synchrony and speculated that synchronous events may be related to activity in primary afferents. Therefore, our primary objective was to investigate synchronous activity in dorsal horn neurons and its possible relation to the activity of primary afferents using *in vitro* preparations from adult mice and larger electrode matrixes which allowed us to increase the sample of neurons simultaneously recorded.

As previously discussed, peripheral inflammation leads to an increase in the backfiring of nociceptive primary afferents (Lin et al., 2000). We have shown recently that this enhanced activity caused by peripheral inflammation develops quickly and persists in the isolated spinal cord demonstrating that the mechanisms underlying backfiring of the afferents are contained within the cord (Vicente-Baz et al., 2022). Therefore, as a secondary objective we aimed at evaluating the impact of a peripheral inflammation on synchronous activity of dorsal horn neurons.

The results obtained in the present investigation are consistent with the hypothesis that bursts of collective activity in populations of dorsal horn neurons are involved in the generation of DRRs and to the central changes induced by peripheral injury.

## Materials and methods

### Animals and ethical approval

Twenty three female CD1 mice (36–50 days old) were used. Animals were bred at the local facility and maintained under a 12–12 h light-dark cycle, humidity of 55 ± 15% and *ad libitum* access to food and water. Surgical procedures were performed under deep anesthesia assessed by the absence of withdrawal reflexes to nociceptive stimuli. After the surgery, the animals remained anesthetized and were killed by decapitation. The experimental design was in agreement with European Union and Spanish Government regulations and comply with the ARRIVE guidelines. All the protocols were approved by the University of Alcalá Ethics Committee and the Community of Madrid Regional Government (PROEX 018/16).

### Carrageenan-induced peripheral inflammation and behavioral testing

The day before spinal cord extraction, treated mice received intra-plantar injections of carrageenan in both hind paws (30 μL, 3% in saline) to enhance the effects of the treatment and to allow recording in both sides of the cord. Paw diameter and mechanical withdrawal threshold were tested in naïve and treated animals just prior to spinal cord extraction. The 50% withdrawal threshold was obtained using calibrated von Frey filaments (0.02–4 g) following the up and down method (Chaplan et al., 1994). Paw diameter was measured using a precision vernier caliper.

### Preparation of spinal cord slices

Preparation of slices has been previously described in detail (Lucas-Romero et al., 2018). Briefly, after deep anesthesia (urethane, 2 g/Kg i.p.), a lumbar laminectomy was performed and the cord dissected and placed in sucrose-substituted artificial cerebrospinal fluid (ACSF) at 4°C. Then, meninges were removed and, in some spinal cords, L4 dorsal root was gently teased to obtain thin rootlets to record individual primary afferents, as previously reported (Pozo-Jimenez et al., 2021). Finally, we obtained a single horizontal slice of about 500 μm thick using a vibratome (Sectioning Systems 1500), which contained the dorsal horn and dorsal roots attached. The slice was pinned down to the Sylgard bottom of the recording chamber, with the cut surface up, and maintained by perfusion with oxygenated ACSF at 22 ± 1°C (composition in mM: NaCl 127, KCl 1.9, KH_2_PO_4_ 1.5, MgSO_4_ 1.3, CaCl_2_ 2, NaHCO_3_ 22, and glucose 10, pH 7.4).

### Dorsal horn extracellular recordings

For the recording of action potentials, we used microelectrode arrays (MEAs) consisting of 32-recording sites distributed across 4 shanks separated by 200 μm (Buzsaki 32-A32, NeuroNexus). With the electrode mounted on a computer-controlled micromanipulator (Patchman NP2, Eppendorf) and placed by the side of the preparation, the level of the Sylgard floor was taken as zero reference. Then we placed the MEA over the L3–L4 border and lowered the electrode deep in the preparation until the last row of sensors was 100 μm below the sectioned border. Then, the electrode position was adjusted with smooth up and down movements to maximize the number of spontaneously active neurons recorded and allowed to stabilize for 30 min prior to start the experiment.

Signals from the MEA were amplified, band pass filtered between 200 and 3 KHz and digitized using RHS2116 amplifier chips and an RHS2000 stimulation/recording controller (Intan Technologies, USA). Data was then stored for offline analysis using Spike-2 software from CED.

Using electrode structure and electrode position within the cord, we estimated recordings to be superficial (laminae I–III) when they were obtained at a distance ≤ 260 μm from the dorsal border of the cord or deep (laminae IV and V) otherwise.

### Dorsal root recordings

The L4 root was placed inside a suction electrode pulled manually from borosilicate capillary tubes. In a few experiments, recordings were obtained from small caliper rootlets carefully teased from the L4 using fine forceps. Rootlets were placed inside micro-suction electrodes pulled with a micropipette puller (P97, Sutter Instruments) and cut down under microscopy supervision to get an external diameter of 30–90 μm. Signals from roots and rootlets were processed with identical settings. In both cases, the signal recorded was amplified (AxoClamp 2B, Axon instruments), AC and DC filtered (Digitimer Ltd., NeuroLog Systems), digitized (RHS2000 stimulation/recording controller, Intan Technologies) and stored for offline analysis.

### Drugs and chemicals

Picrotoxin as well as the components for ACSF were purchased from Sigma Aldrich. A stock solution of picrotoxin in DMSO at 20 mM was prepared and separated in aliquots that were stored at –20°C. For the experiments, a single aliquot was diluted in ACSF at 20 μM and perfused during 30 min to fresh slices after 30 min of pre-drug recording.

### Analysis of activity in the dorsal horn and detection of synchronous events

Spike2 software (Cambridge Electronic Design) was used for data visualization and spike sorting. Sorting of spikes from dorsal horn neurons and primary afferents was performed using principal component analysis based on spike shape and, when possible, on multichannel comparison analysis. After sorting, each isolated spike form, presumably generated by a single neuron, was transferred to a separate channel and a time stamp was created for each spike.

Spontaneous activity from each neuron was classified using a previously defined algorithm (Lucas-Romero et al., 2018) which is publicly available.^1^ This algorithm analyses a spike train and defines its firing pattern according to the regularity of spikes measured by the coefficient of variation and the presence of bursts.

The best procedure tested to detect quasi-synchronous spikes from different neurons (i.e., “population bursts”) started by exporting to MATLAB all channels containing the spike time stamps. This file was treated with an algorithm built in house^2^ to detect population bursts in a semiautomatic mode. The algorithm collapsed time marks from all individual neurons into a single channel and plotted the data as mean firing frequency using the Spike 2 formula in which, for any given event, other events included in the same window (defined by the bin size) were used to calculate the frequency value, defined as:


$$\frac{n-1}{t\,e-t\,l}\,i\,f\,\left(t\,e-t\,l\right)>\frac{t\,b}{2}$$

$$\frac{n}{t\,b}\,i\,f\,\left(t\,e-t\,l\right)\leq \frac{t\,b}{2}$$

Where *tb* is the bin size (*tb* = 0.2 s in our case), *te* is the time of the current event, *tl* is the time of the first event in the time range and *n* is the number of events in the time range. An amplitude threshold was adjusted for each experiment just above baseline firing.

Once the population bursts were detected, we drew the channel as a frequency histogram and created a precise time mark for each burst at the time of maximal frequency. Two hundred ms time windows starting 50 ms before and ending 150 ms after the mark contained the spikes forming the quasi-synchronous event and were used for analysis. For every neuron, we calculated the number of action potentials fired within these windows out of all spikes fired by the neuron along the recording time. These percentages were taken as an index of spike grouping in synchronous events and were used to calculate mean values per class or treatment. The distribution of action potentials within time windows was calculated as well. To this purpose, the population burst window was divided in 5 ms bins and the number of action potentials fired by each neuron in these bins was averaged for all the bursts in the experiment. Mean values per neuron and bin were then analyzed for different sets of neurons.

Cross-correlograms, correlograms, raster plots and waveform averages were obtained using Spike2 software.

In order to assess picrotoxin effects on spontaneous activity of neurons, we compared the mean firing frequency in a 5-min window just prior to drug perfusion with the firing frequency recorded in an identical time window at the end of a 30 min period of picrotoxin superfusion. A neuron was considered sensitive if picrotoxin produced ≥ 70% reduction in its firing frequency. For these experiments, the probability of a neuron to fire within bursts by chance was calculated. We took the total number of action potentials of each single neuron and created random distributions along the respective recording times using the function ‘rand’ from MATLAB. These distributions were then used to calculate the number of spikes that fell within the time limits of each population burst detected. This process was repeated a 100 times using different seeds to obtain averaged random grouping values per neuron.

### Analysis of effective connectivity between dorsal horn neurons and primary afferents

For the analysis of effective connectivity between neurons and primary afferents, we used a machine learning-based tool constructed with the algorithm C5.0 (Quinlan, 1993) that we developed, explained in depth and tested in synthetic and biological circuits in a previously published work (Pozo-Jimenez et al., 2021).

Briefly, the algorithm uses the spike trains of one or several neurons as attributes to predict the behavior of a target (neuron or afferent). The algorithm is trained in a data subset by analyzing the activity in the attribute during the 50 ms preceding each spike of the target. During training, the algorithm builds rules to take decisions and then the predictive value of these rules and decisions are tested in a different data subset. The overall predictive value of the rules created for a target was assessed with a metric called Matthew’s correlation coefficient (Matthews, 1975; Chicco, 2017). This score is calculated from the results of the confusion matrix obtained when the predictions made by the algorithm are compared with actual data during the testing phase. The maximum value for the Matthew’s correlation coefficient (MCC) is 1 and values ≥ 0.18 for single neurons as attributes have been associated to monosynaptic excitatory connections using complex synthetic circuits in which more than one neuron is required to trigger action potentials in the target (Pozo-Jimenez et al., 2021). Therefore, here we used this value of MCC as indicative of a strong functional relation between neurons.

For the present work, we run the algorithm using afferents as target to find individual neurons potentially connected to the afferent in a presynaptic position. Then each individual neuron was used as target to assess the likelihood of a neuron to be post-synaptically located to the afferent.

### Statistics

All data in text and graphs are presented as mean ± standard error of the mean (SEM). Prism 7.0 from GraphPad was used for statistical analysis and generation of graphs. Contingency tables to study differences in proportions were analyzed with Fisher’s exact test and Chi square test. Mann–Whitney and Wilcoxon matched-pairs signed rank tests were used for unpaired and paired comparisons, respectively. Two-way ANOVA was used for multivariate analysis. *Post hoc* analyses were performed using Sidak’s and Tukey’s multiple comparisons test. We provide exact p values where possible. When multiple comparisons were performed we only report the maximum *p*-value for significant differences or minimum *p*-value for non-significant differences.

### Experimental design

The use of MEAs caused difficulties to make an accurate definition of the effective sample size for the study *a priori*. The yield of MEA recordings is highly variable due to the inherent variation in the location of the sensors and in the successful isolation of activity into single identifiable neurons. From our previous experience using these MEAs with 32 sensors we estimated average yield ≈10 neurons per successful electrode positioning. Considering the size of the MEA, it is possible to maintain a sufficient distance between electrode tracks in the rostro-caudal and mediolateral axis to perform up to 9 different tracks from a single preparation (3 mediolateral × 3 rostro-caudal positions), being confident to exclude the possibility of repeated recordings from the same neuron in successive tracks. The depth of the recording sites also constitutes an additional dimension to avoid duplicated recordings. However, due to time limitations during the recording session, most experiments had ≤ 3 tracks per animal. Furthermore, electrode positions with less than 3 well-isolated neurons were discarded for the analysis due to the impossibility to detect synchronous activity. Additionally, in preparations used for pharmacological experiments only data from a single electrode positioning was included in the analysis to ensure that neuronal activity was not modified by the previous application of the drug. The apparent misbalance between the number of naïve and treated mice is explained by the fact that drug application was performed in 9 cords from naïve animals. Three additional preparations from treated mice were also assayed with PTX to confirm in this different condition the results observed in naïve.

Taking into consideration all these determinants, for the study design we estimated that this distribution of animals would yield ≥ 200 well isolated neurons in each condition. This amount of isolated neurons enables to obtain a sufficient number of recordings to find the most common spike train patterns previously described and to allow for statistical comparisons between them.

Our study was directed to study the characteristics of spontaneous spike trains in dorsal horn neurons, the presence of coordinated activity and the existence of differences between naïve and treated mice. For this reason, our statistical comparisons need to be made between individual neurons, considering the whole population, but also segregated by firing pattern since important differences exist in their particular characteristics. However, multiple recordings for the same animal/preparation may entail complications for the statistical analysis due to the presence of non-independent (nested) data. We first tested if neurons recorded from the same animal showed some extent of dependency that may preclude the use of conventional statistical methods (Aarts et al., 2014). To analyze if individual neurons may be considered as independent observations we calculated the intracluster correlation (ICC) for mean firing frequency values in the whole population of neurons studied. ICC was calculated as the variance between animals divided by the total variance (variance between animals and variance between neurons within each animal). An ICC value of 0.155 was obtained, indicating that exits more variability in the firing frequency within (≈84%) than between (≈16%) animals. Since the similarity between neurons within the same animal is low, neuronal behavior can be considered mostly unique and independent of the subject.

To study the relationship between activity in dorsal horn neurons and primary afferents we used the algorithm C5.0 to define the neurons involved and to obtain clues about the direction of the connectivity. This approach was not intended to obtain quantitative data nor to compare differences between naïve and treated mice. For these studies we used 12 animals, but more trials were made in treated (8/12) than naïve mice since previous literature indicate that are more prone to show antidromic activity in the form of action potentials that can be recorded from rootlets (Lin et al., 2000). The procedure developed was technically challenging and successful recordings from afferents were only obtained from 2 out of 4 naïve and 3 out of 8 treated mice. Simultaneous recordings of DHNs with MEAs were obtained in 3 out of 5 of these experiments. Only data from these 3 animals is included in the results shown.

## Results

### General data and characterization of spontaneous neurons

**FIGURE 1:**
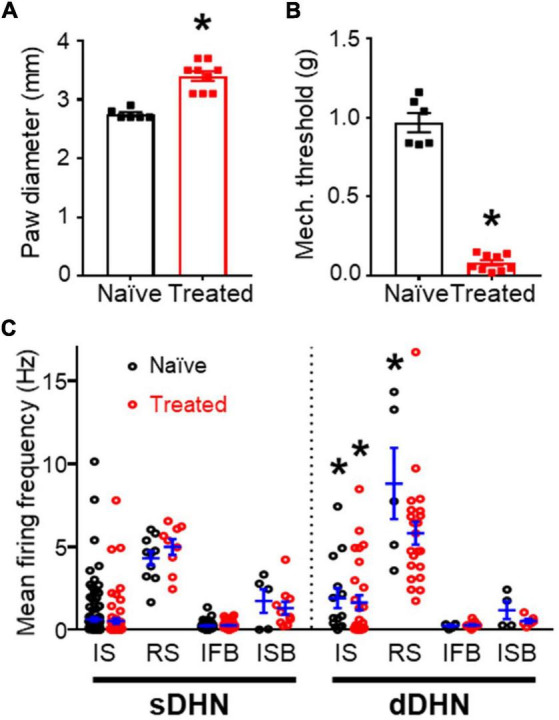
General comparisons between naïve and treated animals. **(A)** Effect of inflammation on paw diameter. **(B)** Effect of inflammation on mechanical withdrawal threshold. Significant differences are marked by * (*p* = 0.0004, Mann–Whitney test for panels **A,B**). **(C)** Mean spontaneous firing frequency in the sample of neurons recorded, which comprises different classes of neurons (for this and subsequent figures: IS, single spike; RS, regular simple neurons; IFB, irregular fast burst; ISB, irregular slow burst) from naïve and treated animals in superficial (sDHN) and deep (dDHN) laminae. There was a significant effect of neuronal class on firing frequency in both superficial [F(3, 335) = 87.34; *p* < 0.0001] and deep areas [*F*(3, 90) = 28.28; *p* < 0.0001; Two-Way ANOVA], no differences were found for treatment and interaction. RS neurons had significantly greater firing frequency than the other types in pairwise comparisons (Tukey’s multiple comparisons *post hoc* test *p* ≤ 0.0004). IS and RS neurons had greater firing frequencies in deep than superficial areas as marked by * (Tukey’s multiple comparisons *post hoc* test, *p* ≤ 0.0093).

The data presented in this report was obtained from 23 adult mice, 8 of which had been treated with an intra-plantar injection of carrageenan 20 h prior to spinal cord extraction and the remaining 15 animals were age matched naïve mice. Immediately before cord extraction, the intra-plantar injections had caused a significant increase in paw diameter and a significant decrease in mechanical withdrawal threshold (Figures 1A,B).

Spontaneous activity was recorded from 249 naïve neurons and 215 neurons from treated animals (see Table 1). Neurons were assigned to one of 8 classes based on their patterns of spontaneous activity as previously reported (Lucas-Romero et al., 2018). There were four classes of irregular firing patterns (single spike or IS, fast burst or IFB, slow burst or ISB, and mixed burst or IMB) each one having a regular firing counterpart (RS, RFB, RSB, and RMB). IS, RS, and IFB neurons were the most commonly recorded classes (see Table 1). The composition of the neuronal sample considering treatment, neuronal class and recording location was complex and unbalanced which posed some constrains to the analysis of results. Recordings from superficial locations were more abundant in naïve (204/240) than in the treated group (143/215; *p* < 0.0001, Fisher’s exact test). However, neurons with regular firing patterns were more commonly found in deep (35/108) than in superficial laminae (20/347; *p* < 0.0001, Fisher’s exact test). Furthermore, considering the subsample of superficially located recordings, the proportion of IS vs. IFB neurons was more balanced in the treated group (66 IS vs. 58 IFB) than in the naïve group (129 IS vs. 56 IFB; *p* = 0.0039, Fisher’s exact test) (see Table 1).

**TABLE 1:** Numbers of neurons recorded per treatment, class and depth.

		Irregular firing					Regular firing				
Treatment	Location	IS	IFB	ISB	IMB	Total	RS	RFB	RSB	RMB	Total
Naïve	Superficial	129	56	5	3	**193**	10	1	0	0	**11**
	Deep	14	6	4	1	**25**	5	0	5	1	**11**
Inflammation	Superficial	66**	58*	10	0	**134**	9	0	0	0	**9**
	Deep	28	13	6	1	**48**	22	0	2	0	**24**

Summary of the total number of neurons included in each condition arranged by their location in the dorsal horn and their firing pattern. Statistically significant differences between naïve and treated conditions are marked by asterisks (Fisher’s exact test; *p = 0.146; **p = 0.002).

Firing frequency was strongly related to neuronal class (see Figure 1C). RS neurons showed the largest mean firing frequency under all conditions. Deep IS and RS neurons showed higher firing frequencies than the equivalent superficial neurons. The inflammatory treatment did not produce significant changes in firing frequency per neuronal class.

### Synchronous events

**FIGURE 2:**
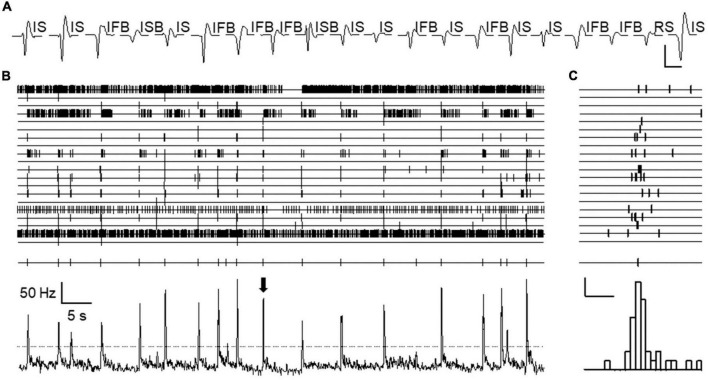
Population bursts observed during spontaneous activity. **(A)** Shows sorted spike forms from an experiment. Twenty spike forms were isolated, presumably generated by 20 different neurons. Each trace is an average of all spikes belonging to each isolated unit during the whole length of the experiment. Patterns of spontaneous activity are displayed for each spike using acronyms defined in text. Calibration bars 0.2 mV and 2 ms. Channels 1–20 (from top to bottom) in panel **(B)** show time stamps for each action potential fired by each neuron as sorted in panel **(A)** (from left to right) recorded during an approximate period of 1 min. All time stamps were collapsed into a single channel and represented as mean frequency (bottom channel). Peaks in this channel show the occurrence of “population bursts.” Threshold for population burst detection in this experiment is indicated by the discontinuous line. Time marks for bursts are shown just above. **(C)** Shows in expanded time base, the firing of each neuron during a single population burst (marked by arrow in panel **B**). The lower panel shows a combined frequency histogram and just above it, the burst mark placed at the time of maximum firing frequency. Calibration bars 2 action potentials and 100 ms.

It was obvious from visual inspection of the recordings that spontaneous activity distributed in a non-homogeneous fashion along the time axis. Action potentials from different neurons tended to cluster together in narrow time windows and this occurred without any apparent periodicity (see Figure 2). We called these events “population bursts.” Population bursts were identified and time marked at maximum firing frequency as explained in methods.

The occurrence of population bursts was detected in 12/15 experiments using naïve animals and in 8/8 experiments with treated mice. This allowed analyzing the characteristics of population bursts in 17 recording locations from naïve and another 17 locations from the treated group. For the following analysis, we only included neurons from electrode locations in which population bursts were detected (186 naïve and 199 treated).

**FIGURE 3:**
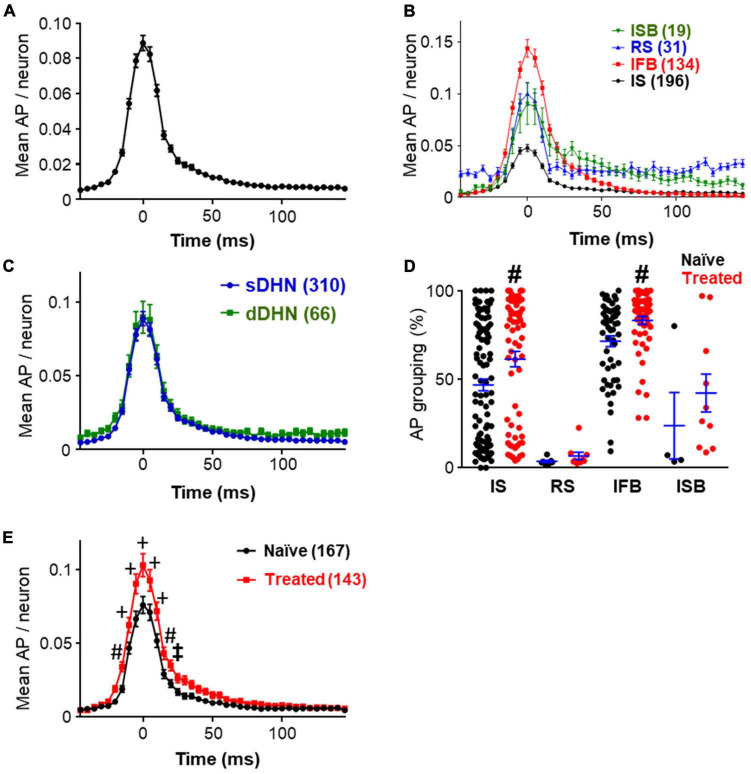
Structure of bursts. **(A)** Shows the prototypical shape of the population bursts recorded showing the mean number of spikes that each neuron fired during the burst window in 5 ms bins. Data include all the neurons from tracks in which population burst were detected, pooling together data from naïve and treated, deep and superficial (*n* = 385). **(B)** Graph shows the shape of the population bursts recorded from different neuronal classes as indicated (labeled by their acronyms). All neurons were included as in panel **(A)**, the number of neurons are indicated in brackets. Supplementary Figure 1 shows paired comparisons and detailed statistical differences. **(C)** Population burst forms obtained segregating neurons by depth of recording (superficial dorsal horn neurons, sDHN; deep dorsal horn neurons, dDHN; number of neurons in brackets). Data from both experimental groups were pooled together. Statistically significant differences were found for recording depth [*F*(1, 15334) = 24.74; *p* < 0.0001] and time point [*F*(40, 15334) = 104.2; *p* < 0.0001], but no significant interaction was found. Point by point comparisons with Sidak’s multiple comparisons were found not significant. **(D)** Percentage of action potentials falling within population burst windows including neurons from superficial laminae only. Neurons from different groups (naïve in black; treated in red) and neuronal classes are shown. Note that IS and IFB neurons have greater grouping values than other neurons. The grouping of IS and IFB neurons was significantly increased by treatment (*p* ≤ 0.0039; Mann–Whitney test). For panels **(D,E)**, statistically significant differences are marked as: ^‡^*p* < 0.05; ^#^*p* < 0.01, and ^+^*p* < 0.001. **(E)** Graph showing differences in the shape of population burst as obtained from superficial neurons in naïve and treated conditions (number of neurons indicated in brackets). A two-way ANOVA analysis shows statistically significant differences for treatment [*F*(1, 308) = 7.992; *p* = 0.0050] and time point [*F*(40, 12320) = 230.2; *p* < 0.0001], and a significant interaction between factors [*F*(40, 12320) = 6.037; *p* < 0.0001]. Sidak’s multiple comparisons test following ANOVA found differences between treated and naïve groups at several time points as indicated.

Pooling together the data on mean spikes per neuron and bin, we obtained graphs that characterize the shape of the population bursts as show in Figure 3, which summarizes the findings reported in this section. Considering all neurons recorded, deep and superficial, under naïve and treated conditions (Figure 3A), the emerging picture of a population burst is a fast increase in firing frequency starting ∼25 ms before the burst mark, followed by a slower return to baseline which lasted ∼75 ms. Breaking down the components of this population bursts by neuronal class we found that all classes of neurons contributed although to different extents (see Figure 3B and Supplementary Figure 1). Two-way ANOVA analysis comparing the curves showed that IFB neurons started to fire earlier than the other types (*p* ≤ 0.04, Tukey’s multiple comparison test), whereas ISB neurons had a slower return to baseline (*p* ≤ 0.03, Tukey’s multiple comparison test). Peak firing frequencies were also greater for IFB neurons as shown in Figure 3B (*p* < 0.0001). RS neurons increased their firing frequency during the bursts as well, but from a higher baseline.

Breaking down the results by recording location, we found that neurons in deep and superficial laminae were both involved in population bursts (Figure 3C). The graphs obtained for deep and superficial bursts were very similar. The slightly elevated baseline in the curve from deep neurons was due to the large proportion of RS neurons recorded in deep layers. However, the point by point difference between the two graphs was not statistically significant (Sidak’s multiple comparisons test *p* > 0.98).

Comparing naïve and treated groups, no difference was found in population burst frequency (0.173 ± 0.016 Hz vs. 0.178 ± 0.013 Hz, *p* = 0.7596, Mann–Whitney test). The effects of the inflammatory treatment on population bursts were studied using the data from superficial recordings only (167 neurons from naïve and 143 from treated). Recordings from deep neurons were excluded from the following analysis due to the unbalanced composition of the sample obtained (see Table 1).

The mean firing frequency of naïve and treated neurons in superficial dorsal horn was similar (0.72 ± 0.15 for naïve vs. 0.76 ± 0.13 for treated, *p* = 0.2096, Mann–Whitney test). The grouping of action potentials in bursts under naïve and treated conditions is shown in Figure 3D. The mean grouping of action potentials in population bursts increased from 51.7 ± 2.6% in naïve to 65.5 ± 2.8% in treated condition (*p* < 0.0001, Mann–Whitney). All neuronal classes showed larger grouping values under the treated condition; however, significant differences were only obtained for IS and IFB neurons (see Figure 3D).

The increased grouping of action potentials in bursts under the treated condition resulted in significant changes in the shape of the burst as reflected in Figure 3E. A two-way ANOVA comparison of the graphs obtained for naïve and treated conditions detected a significant difference between conditions. Point by point comparison between graphs indicate that population bursts from the treated group had more action potentials at the raising, at the peak and at the decaying phases than the naïve group.

### Relation of population bursts to backfiring of primary afferents: effects of GABA_A_ receptor blockade

From previous observations, we suspected a relation between population bursts and activity in primary afferents in the form of dorsal root potentials or dorsal root reflexes. Since blockade of GABA_A_ receptors has a potent inhibitory effect on dorsal root reflexes (Levy et al., 1971), we expected that blockade of this receptor altered the structure of population bursts as well.

**FIGURE 4:**
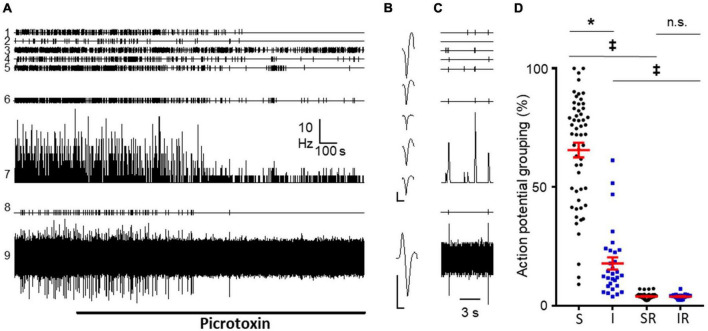
Effect of picrotoxin perfusion on DRRs and population bursts. **(A)** Recordings from an experiment from a naïve mice showing effects of picrotoxin (20 μM) on five different dorsal horn neurons (channels 1–5). Channel 6 correspond to population burst marks; channel 7 shows mean frequency graph for neurons in 1–5 considered together; channel 8 shows time stamps for action potentials from a primary afferent; channel 9 is the corresponding original recording from the afferent. Note the potent inhibitory effect of picrotoxin on spikes generated by the afferent. In contrast, picrotoxin inhibited only partially the activity of dorsal horn neurons and reduced frequency and amplitude of bursts. **(B)** Spike forms from dorsal horn neurons (1–5 in panel **A**) and from the primary afferent (9 in panel **A**). Calibration bars 0.2 mV and 1 ms. **(C)** Shows three population bursts in an expanded time base prior to PTX perfusion. **(D)** Graph shows the actual grouping of action potentials in population bursts for neurons sensitive (S) and insensitive (I) to picrotoxin (* for *p* < 0.0001, Mann–Whitney test). Columns 3 and 4 show the expected grouping values from random distribution of spikes in sensitive (SR) and insensitive (IR) neurons. Note how actual grouping values are higher than expected by chance even in the group of neurons insensitive to picrotoxin (^‡^for *p* < 0.0001, Wilcoxon matched-pairs signed rank test). No significant differences were found between randomized groups (*p* = 0.6857; Mann–Whitney test).

To test this prediction, we perfused picrotoxin (PTX, 20 μM) for a prolonged time (30 min) to 9 naive preparations including 96 neurons (91 of them superficial). In 7 out of 9 preparations it was possible to detect population bursts with a mean frequency of 0.19 ± 0.02 Hz. The application of PTX abolished population bursts in one experiment and strongly depressed their frequency in 6 experiments in which low amplitude bursts were still detected (0.03 ± 0.01 Hz; *p* = 0.002, Wilcoxon matched-pairs signed rank test, see Figures 4A–C). In one experiment (shown in Figure 4A) it was possible to record the simultaneous activity from a single primary afferent during perfusion of PTX and this activity was abolished as well.

Considering individual neurons, PTX inhibited firing in 27/32 irregular fast burst neurons and 29/61 irregular single spike neurons, being the former class more sensitive (*p* = 0.0004, Fisher’s exact test). The remaining neurons were considered insensitive to PTX. This sample included 8 IS neurons that increased their firing to > 160% of control values. Three IS neurons that did not change mean firing rate, became regular (RS) after perfusion of the blocker.

Comparing neurons sensitive and insensitive to PTX, the former presented a greater grouping of action potentials in population bursts prior to perfusion of the antagonist (65.6 ± 3.1% vs. 17.8 ± 2.6%; *n* = 55 and 30; *p* < 0.0001 Mann–Whitney test; see Figure 4D). However, neurons insensitive to PTX had grouping values higher than expected by random (see Figure 4D).

Similar results were obtained in 3 additional experiments using cords from treated animals. Neurons with large grouping values, mainly IS and IFB were inhibited by PTX.

### Relation of population bursts to backfiring of primary afferents: Timing relation between action potentials originated at primary afferents and dorsal horn neurons

To record activity from afferents, we first included whole dorsal roots in suction electrodes but this approach was unsuccessful at identifying either sub- or suprathreshold events. Therefore, we recorded from teased rootlets using micro suction electrodes. With this new approach, reliable and stable recordings of spikes from primary afferents combined with MEAs were obtained in three experiments (1 naïve and 2 treated) out of 12 attempts. Sorting of recordings obtained from rootlets in these experiments revealed 8 different spike forms presumably originated at single afferents (1 from naïve and 7 from treated). Under the present experimental conditions, it was not possible to identify fiber types.

**FIGURE 5:**
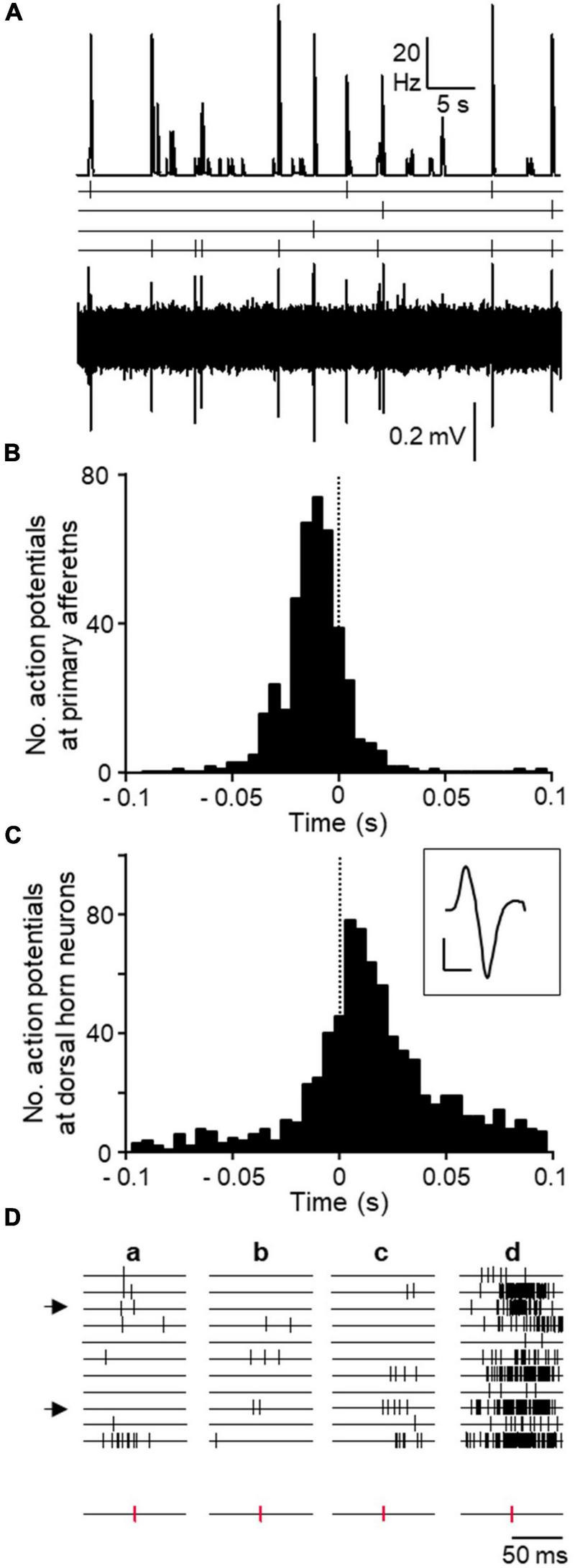
Correlation between DRRs and spontaneous spikes from dorsal horn neurons. **(A)** Original recordings showing the coincidence in time between population bursts (top trace) and action potentials recorded from 4 isolated primary afferents (time stamps and original recording below). This experiment was performed on tissue from a treated animal. **(B)** Cross-correlograms from the same experiment showing the distribution of action potentials from all 4 primary afferents around the population bursts mark (time 0 and dotted line). **(C)** Cross-correlograms from a different experiment showing action potentials from dorsal horn neurons taking action potentials in one afferent as trigger (time 0 and dotted line). Averaged spike form from afferent in inset (calibration bars 0.2 mV and 1 ms). **(D)** Time stamps of action potentials from 11 dorsal horn neurons around three occurrences of single action potentials in an afferent (a–c, time stamps for action potentials in afferent separated at bottom). Note the lack of patterned activity in neurons. The last column (d) shows cumulative firing of each neuron in relation to action potentials in the afferent. Arrows at left mark two neurons identified as putative presynaptic to the firing of the afferent as explained in text.

Correlation between bursts and action potentials in primary afferents was obvious from visual inspection of recordings (Figure 5A). Action potentials recorded from primary afferents reached a maximum during the building up of the population burst. The correlation was clearly represented in cross-correlograms using the burst mark as trigger (see representative example Figure 5B). As shown, action potentials from afferents tended to occur preceding the burst mark. The distribution of spikes fired by dorsal horn neurons using action potentials in afferents as trigger were studied as well and a representative example is shown in Figure 5C. There is a clear grouping of action potentials in dorsal horn neurons around the firing time of the afferent. Firing of action potentials in afferents were preceded and followed by spikes in dorsal horn neurons.

We made a visual inspection of individual action potentials from afferents to check for possible regularities or repeated sequences of spikes in dorsal horn neurons that may have been necessary to trigger an action potential at the afferent. As shown in Figure 5D, associated to the firing of an individual afferent, some dorsal horn neurons tended to fire more often than others did. However, no obvious regularities were found for any of the afferents.

### Effective connectivity between dorsal horn neurons and primary afferents

**FIGURE 6:**
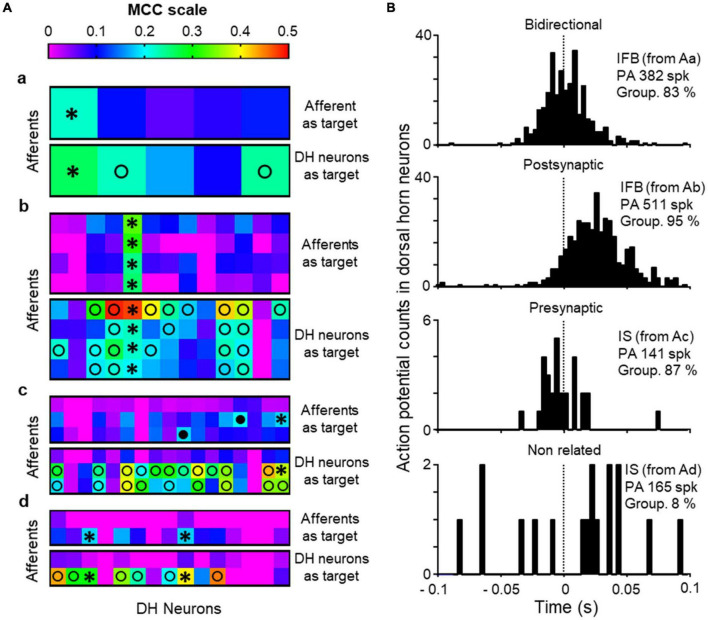
Effective connectivity analysis. **(A)** Shows heat maps for effective connectivity as estimated with the MCC (color code at top). Values of MCC ≥ 0.18 were considered as putative monosynaptic connections. For each of the recordings performed (a–d) the upper map shows the likelihood of a dorsal horn (DH) neuron to be presynaptic to the afferent (afferent as target) and the bottom panel the likelihood of a DH neuron to be post-synaptic to the afferent (DH neuron as target). Black circles mark DH neurons considered as putative presynaptic to an afferent, open circles mark DH neurons putative post-synaptic to afferent neurons and * stand for bidirectional connections between DH neurons and afferents. **(B)** Correlograms between the firing of single afferents (time 0 and dotted line) and neurons showing different types of relation between them. The information in insets include the type of neuron, in parenthesis correspondence with recordings in panel **(A)**, number of action potentials of the primary afferent used to build the correlogram (PA) and the grouping of action potentials in population bursts for the neuron.

Further to the previous analysis, we wanted to find whether the neurons recorded were pre- or post-synaptically associated with the afferents. To this end, the effective connectivity between the 8 primary afferents and the 50 dorsal horn neurons recorded was studied applying the AI algorithm described in methods. Summarized results are shown in Figure 6. One of the afferents was recorded from a naïve cord and studied in conjunction with 5 different dorsal horn neurons (Figure 6Aa). In a second experiment, 4 afferents and 13 dorsal horn neurons were recorded (Figure 6Ab). In the last experiment, 3 afferents were recorded simultaneously with 17 dorsal horn neurons in a first electrode positioning (Figure 6Ac). Then, the electrode matrix was repositioned in a new location that allowed recording of 15 additional dorsal horn neurons while retaining the 2 first afferents (Figure 6Ad).

Using the afferents as target, we obtained MCC values ≥ 0.18 for 7 dorsal horn neurons (mean 0.21, range 0.18–0.33) which were considered putative presynaptic neurons. These neurons showed a mean firing frequency of 0.35 ± 0.07 Hz and a mean grouping of action potentials in population bursts of 76.8 ± 5.0%. Five of them were of the irregular fast burst type and the remaining 2 were irregular single spike. In one case, several dorsal horn neurons with high MCC were related to the same afferent (two IS neurons in Figure 6Ac and two IFB in Figure 6Ad). In another experiment, one single IFB neuron had high MCC values related to the 4 afferents recorded (Figure 6Ab).

Additionally, the AI algorithm was run for each individual dorsal horn neuron as target and the afferents as attribute. For 33 dorsal horn neurons we found high MCC values (> 0.18) suggesting a post-synaptic position with respect to the afferent. Six out of the 7 putative presynaptic neurons were included in this group suggesting bidirectional connections between afferents and neurons. The remaining putative post-synaptic neurons were IS (*n* = 12) and IFB (*n* = 15). Post-synaptic neurons showed a mean firing frequency of 0.21 ± 0.03 Hz and a high grouping of action potentials in population bursts (mean 85.2 ± 2.0%). Sixteen more neurons (13 IS, 1 IFB, 1 ISB, and 1 IMB) showed low MCC values suggesting a more distant relation with the afferents. These had a firing frequency of 0.60 ± 0.32 Hz and a mean grouping index of 39.6 ± 7.3% (significantly lower than dorsal horn neurons closely related to the afferents, *p* < 0.0001, Mann–Whitney test).

In summary, our analysis shows strong effective connectivity between 7/8 afferents and 34/50 dorsal horn neurons of which 7 were putative presynaptic and 27 putative post-synaptic with respect to one or more afferents. For one afferent it was not possible to find dorsal horn neurons with close pre or post-synaptic relation according to MCC values. Figure 6B shows correlograms between single afferents and single dorsal horn neurons, which are representative of the different types of relation between them.

## Discussion

Extracellular recordings of dorsal horn neurons with MEAs allowed the recording of spontaneous activity that followed identifiable patterns. The spontaneous activity patterns described here for dorsal horn neurons have been reported *in vivo* and may contribute to maintain the excitability of sensory and motor circuits (Sandkühler and Eblen-Zajjur, 1994; Lucas-Romero et al., 2018; Bandres et al., 2021). Considering the location of the recording site, we found differences in the distribution of firing patterns and mean firing frequencies between superficial and deep areas (Figure 1C), in agreement with data recently obtained *in vivo* (Bandres et al., 2021). As previously proposed the most plausible origin of spontaneous activity is the intrinsic rhythmicity shown by regular firing neurons which are insensitive to synaptic blockade but sensitive to blockade of ion channels that mediate regularity (Li and Baccei, 2011; Lucas-Romero et al., 2018).

The first original observation reported here is the existence of coordination between the firing of different dorsal horn neurons to produce synchronous events that we have called population bursts. These fast events (∼100 ms) occurred at irregular intervals (see Figure 2B). Neurons of all classes participated of the events to different extents. The roles of IFB and some IS neurons were remarkable because they showed a high proportion of their action potentials within these events (Figure 3D). In addition, they are the most abundant neuronal types, which means that many of the action potentials in population bursts originated at these neurons. It is interesting to note that neurons in superficial and deep laminae were involved in population bursts. Even more interesting, the shape of the events underwent subtle but significant changes in cords from animals that had suffered an inflammation. Synchronous activity has been reported among neurons in many regions of the CNS where it may be part of a processing strategy of information used by local circuits (see for example Cossart et al., 2003).

A second important and novel observation reported here is the association between population bursts from dorsal horn neurons and activity in primary afferents. We show this relation using different approaches. Firstly, we show the strong disrupting effect caused by the blockade of GABA_A_ receptors on the organization of population bursts (Figure 4). Since the activity of primary afferents is very sensitive to PTX, we reasoned that this should have an impact on population bursts as the experimental observations corroborated. Although this may suggest that the firing of afferents was the origin of the bursts, it must be noted that some small amplitude bursts were detected after PTX and that neurons insensitive to PTX showed grouping of action potentials greater than expected by chance. Also interesting to note, IFB neurons were particularly sensitive to the blockade of GABA_A_ receptors. Alternative explanations are hard to envisage, however, GABA_A_ activation may have an excitatory effect on neurons with strong rebound excitation after inhibition (Reali and Russo, 2013).

In a second approach, we studied the temporal sequence of events to discover that the backfiring of action potentials in primary afferents occurred at a time when the population bursts were in their rising phase (Figure 5). That is, some action potentials from dorsal horn neurons preceded the backfiring of the afferents and others followed. This observation is consistent with the previous one on the effects of PTX and suggests that some dorsal horn neurons triggered action potentials in primary afferents that were conducted antidromically to the recording site and orthodromically, into other branches, to excite other dorsal horn neurons. The evidence on the temporal relation of these events that we provide is based on few observations due to the technical difficulties involved. However, the observations were very consistent internally, all of them pointing in the same direction. Since primary afferents deliver excitation to dorsal horn neurons, blockade of inhibitory receptors caused a paradoxical inhibition of spontaneous activity among a majority of dorsal horn neurons. A concern relative to these experiments is the fact that the backfiring was detected at a distance from the site of origin of the action potentials, presumably, the afferent’s terminals. However, this distance is small (≤ 2.5 mm) and the maximal estimated error for the slower conducting afferents was ∼5 ms, which does not change the interpretation of the data.

**FIGURE 7:**
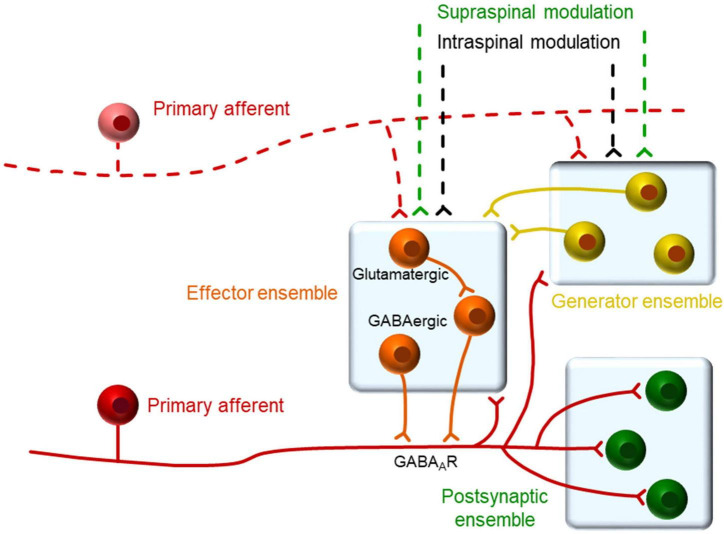
Proposed organization of neurons in relation to primary afferents. We propose that dorsal horn neurons within a single segment may belong to one of three functional types as defined by their relation to the primary afferents. A generator ensemble, formed by spontaneously active neurons, which provide synaptic input to other neurons maintaining the excitability of the system. An effector ensemble formed by neurons in close apposition to the afferent responsible for the actual generation of PAD. A third set of neurons that are excited by the afferent and form a heterogeneous group including projection neurons and interneurons that may belong to the previous ensembles. The activity of this system may be modulated by spinal and supraspinal inputs in the behaving animal.

The relation between single afferents and single neurons was further studied with the use of an AI algorithm that allowed estimating the functional connectivity between the two different elements (Figure 6). The outcome of this analysis reinforces the previous view that some presynaptic neurons triggered action potentials in the afferents and these, in turn, triggered the firing of post-synaptic neurons. The algorithm has been tested in complex synthetic circuits with good results (Pozo-Jimenez et al., 2021) and it is likely to detect close functional connectivity such as that produced by monosynaptic connections. Putative presynaptic neurons detected were of the IFB and IS types, which again, suggest a particularly relevant role for these neurons in the generation of backfiring and population bursts. Putative post-synaptic neurons were also IFB and IS neurons and the role of other neuronal types will require further investigation to increase the number of observations. It is interesting that some neurons were detected as having a bidirectional relation to the afferents. Although this finding is rather puzzling, previous observations have highlighted the possibility of bidirectional connectivity between dorsal horn neurons and primary afferents mediated by glomeruli or other longer pathways (Ribeiro-Da-Silva and Coimbra, 1982; Hughes et al., 2012). The schematic diagram shown in Figure 7, contains our proposed model circuit involved in the observed synchronous events and their relation to dorsal root reflexes.

IFB neurons (as well as a subpopulation of IS neurons) appear to be central elements to the events described. Despite their clear electrophysiological signature, there are few references to them in the literature. Rojas-Piloni et al. (2007) reported these neurons in the superficial laminae of anaesthetized rats and related them to nociceptive processing. These neurons produce a fast burst of action potentials on depolarization and rebound excitations after hyperpolarizing pulses mediated by cationic H and calcium T currents, traits that shape their brisk firing behavior (Rivera-Arconada and Lopez-Garcia, 2015; Roza et al., 2016). The present analysis situates these neurons closely connected to the afferents in pre and post-synaptic positions. It seems plausible that these neurons are interposed between afferents to facilitate that firing in one afferent produces depolarization of neighboring afferents.

The third and last main observation obtained in the present study concerns the effects that a peripheral inflammation has on spontaneous activity and population bursts as recorded from superficial laminae. A comparison between the spontaneous behavior of dorsal horn neurons in naïve and treated animals reveals that firing frequencies of individual neurons and frequency of population bursts do not change significantly. In contrast, the grouping of action potentials in population bursts of treated animals increases significantly for the principal neuronal types involved indicating an enhanced synchrony. This, in turn, generated significant changes on the shape of the population burst (Figure 3E). Changes were detected in the rising phase, which may increase the likelihood of backfiring in the afferents, as well as in the peak and decaying phases reflecting a greater activation of post-synaptic neurons. Both of these predictions from our interpretation have been proved experimentally. Inflammatory treatment has been shown to increase the production of dorsal root reflexes *in vivo* and *in vitro* (Lin et al., 2000; Vicente-Baz et al., 2022) as well as to potentiate spontaneous activity in dorsal horn neurons (Zain and Bonin, 2019). This interpretation of results places firing synchrony at the center of the mechanisms mediating central sensitization.

In summary, the evidence provided supports that PAD and backfiring is a circuit-mediated phenomenon in which activity of a pool of presynaptic neurons (many of them of the IFB type) controls the excitability of terminals by spatial and temporal integration of synaptic inputs. Greater synchrony during sensitized states will increase the chance of exciting the afferents over firing threshold causing backfiring and spreading excitation to post-synaptic neurons.

## Data availability statement

The raw data supporting the conclusions of this article will be made available by the authors, without undue reservation.

## Ethics statement

The animal study was reviewed and approved by the Ethics Committee for research and animal experimentation of the University of Alcalá.

## Author contributions

JL-R performed the experiments and programmed the analysis software. JL-R and IR-A analyzed the data. JL-G coordinated the work and wrote the original manuscript. All authors conceived this work, and revised and contributed to the final version of the manuscript.

## Footnotes

1
https://github.com/Lucas-Romero-J/App_Spike_Train_Analysis

2
https://github.com/Lucas-Romero-J/Population_burst_detector
