# Supplementary material for: Synchronous firing of dorsal horn neurons at the origin of dorsal root reflexes in naïve and paw-inflamed mice
Source: Front Cell Neurosci. 2022 Sep 23;16:1004956. doi: 10.3389/fncel.2022.1004956 (PMC9539274; doi:10.3389/fncel.2022.1004956)
Supplement: Supplementary file 1 [file Data_Sheet_1.PDF]

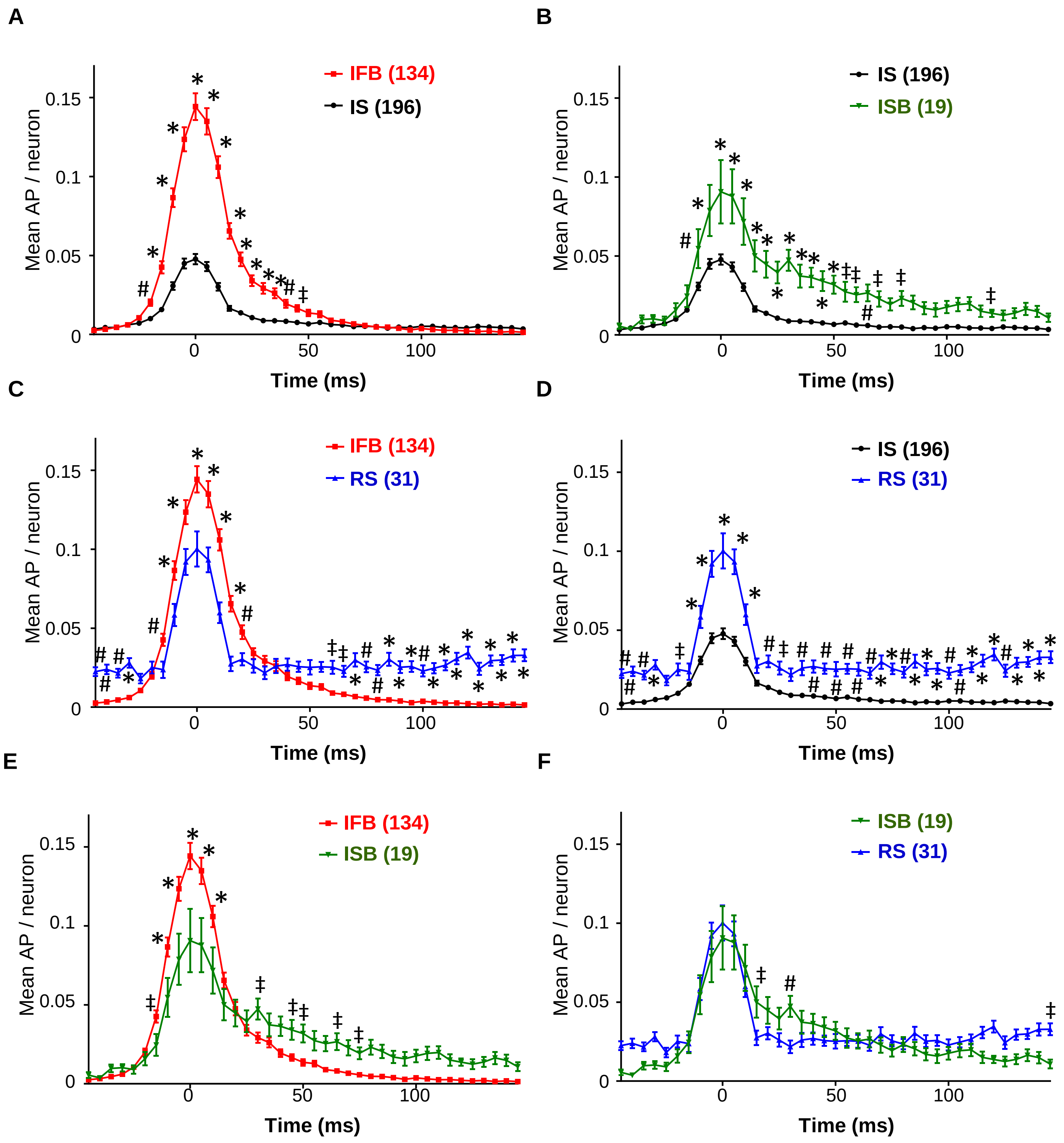

Supplementary figure 1. Contribution of the different neuronal classes to population bursts

Graphs show distribution of spikes in population bursts segregated by neuronal classes as indicated by their acronyms. Data from naïve and treated mice are pooled together and the number of neurons are indicated in brackets. Pairwise comparisons were made between the different classes using two-way ANOVA. Significant differences for class ($F (3, 376) = 37.08$; $p < 0.0001$), time ($F (40, 15040) = 153.7$; $p < 0.0001$) and interaction ($F (120, 15040) = 39.05$; $p < 0.0001$) were obtained. Statistical significance between individual data points was analyzed with a post *hoc* Tukey's multiple comparisons test. Differences are indicated in figures as follows: ‡ $p < 0.05$; # $p < 0.01$ and + $p < 0.001$.
